# High-Gamma Activity Is Coupled to Low-Gamma Oscillations in Precentral Cortices and Modulates with Movement and Speech

**DOI:** 10.1523/ENEURO.0163-23.2023

**Published:** 2024-02-09

**Authors:** Jeffrey Z. Nie, Robert D. Flint, Prashanth Prakash, Jason K. Hsieh, Emily M. Mugler, Matthew C. Tate, Joshua M. Rosenow, Marc W. Slutzky

**Affiliations:** ^1^Southern Illinois University School of Medicine, Springfield 62794, Illinois; ^2^Departments of Neurology, Northwestern University, Chicago 60611, Illinois; ^3^Neurological Surgery, Northwestern University, Chicago 60611, Illinois; ^4^Physical Medicine & Rehabilitation, Northwestern University, Chicago 60611, Illinois; ^5^Neuroscience, Northwestern University, Chicago 60611, Illinois; ^6^Department of Neurosurgery, Neurological Institute, Cleveland Clinic Foundation, Cleveland, Ohio; ^7^Shirley Ryan AbilityLab, Chicago 60611, Illinois; ^8^Department of Biomedical Engineering, Northwestern University, Evanston 60201, Illinois

**Keywords:** ECoG, gamma, LFPs, movement, phase–amplitude coupling, speech

## Abstract

Planning and executing motor behaviors requires coordinated neural activity among multiple cortical and subcortical regions of the brain. Phase–amplitude coupling between the high-gamma band amplitude and the phase of low frequency oscillations (theta, alpha, beta) has been proposed to reflect neural communication, as has synchronization of low-gamma oscillations. However, coupling between low-gamma and high-gamma bands has not been investigated. Here, we measured phase–amplitude coupling between low- and high-gamma in monkeys performing a reaching task and in humans either performing finger-flexion or word-reading tasks. We found significant coupling between low-gamma phase and high-gamma amplitude in multiple sensorimotor and premotor cortices of both species during all tasks. This coupling modulated with the onset of movement. These findings suggest that interactions between the low and high gamma bands are markers of network dynamics related to movement and speech generation.

## Graphical abstract

**Figure:**
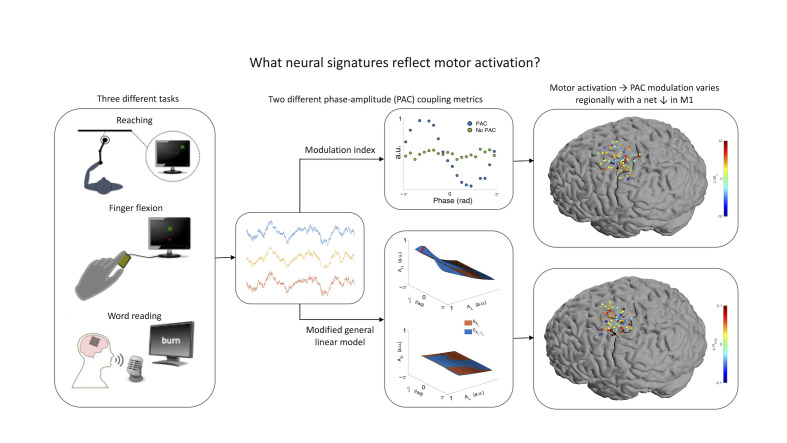
Visual abstract

## Significance Statement

Planning and executing motor behaviors requires coordinated neural activity among different brain regions. Activity in the low-gamma and (to a lesser extent) high-gamma frequency bands is thought to reflect neural information transfer among brain regions across many different behavioral contexts. In monkeys and humans performing different motor behaviors, we found phase–amplitude coupling, a marker of coordinated neural activity, between low-gamma phase and high-gamma amplitude in motor regions. Further, this coupling modulated with the onset of the behavior. This provides insight into underlying network dynamics fundamental to motor control and provides an additional tool for fundamental investigation of cross-area communication in many behavioral contexts and neuropsychiatric conditions.

## Introduction

Local field potentials (LFPs) are generated largely by the ensemble postsynaptic activity of populations of neurons and reflect underlying network dynamics (Buzsáki et al., 2012). Traditionally categorized into several canonical frequency bands, modulation of activity in the theta (*θ*, 4–8 Hz), mu/alpha (*μ*/*α*, 8–13 Hz), beta (*β*, 13–30 Hz), and gamma (*γ*, 40–200 Hz) bands is linked to a wide range of brain functions, such as language perception and production (Crone et al., 2001b; Towle et al., 2008; Flinker et al., 2015) and movement and force production (Pfurtscheller et al., 1996, 2003; Crone et al., 1998; Pogosyan et al., 2009; Engel and Fries, 2010; Igarashi et al., 2013; Brinkman et al., 2016; Flint et al., 2016, 2017). Moreover, studies have demonstrated correlations between LFPs and neuronal spiking (Fries et al., 2001; Chalk et al., 2010; Igarashi et al., 2013; Hyafil et al., 2015) and between LFPs of different frequencies (Canolty et al., 2006; Voytek et al., 2010), the latter case being termed cross-frequency coupling (CFC).

The rhythmicity of LFP oscillations offers an elegant potential mechanism for coordinating neural activity over a wide range of spatial and temporal scales; thus, LFPs are hypothesized to have functional roles by influencing neural activity (Deans et al., 2007; Canolty et al., 2010; Engel and Fries, 2010; Fröhlich and McCormick, 2010; Anastassiou and Koch, 2015; Fries, 2015; Khanna and Carmena, 2017; Pinotsis et al., 2023). In particular, the *γ* band has received substantial attention due to consistent observations of event-related modulations in *γ* band activity and synchronization over a wide range of behaviors and cortical regions (Crone et al., 1998, 2006; Fries, 2009). Although variably defined in the literature (Buzsáki and Schomburg, 2015), in neocortex, the *γ* band is really two distinct bands, low gamma (L*γ*, variably defined but here defined as 40–50 Hz) and high gamma (H*γ*, 70–200 Hz). Oscillations mostly within the L*γ* band are theorized to have an important role in neural communication (Fries, 2009, 2015), whereas H*γ* activity is nonoscillatory, broadband activity, traditionally considered a proxy for ensemble spiking activity (Ray et al., 2008; Manning et al., 2009; Jia and Kohn, 2011; Ray and Maunsell, 2011; Miller et al., 2012; Donoghue et al., 2020).

Movement execution involves coordinated neural activity within higher-order motor cortices (premotor and posterior parietal areas), subcortical nuclei, and cerebellum, and the sensorimotor cortices [primary motor (M1) and primary somatosensory (S1)]. Hypothesized as a marker of coordinated neural activity and information transfer within and between cortical networks (Canolty and Knight, 2010), CFC describes the interactions between LFPs of different frequencies. One type of CFC is phase–amplitude coupling (PAC), in which the amplitude of higher frequency activity varies with the phase of a lower frequency rhythm. Many methods have been developed (Canolty et al., 2006; Tort et al., 2010; Nadalin et al., 2019) and used to detect different types of PAC between several different frequency band pairs during motor behaviors in animals and humans without and with motor disorders (Canolty et al., 2006; Miller et al., 2012; Yanagisawa et al., 2012; De Hemptinne et al., 2013; Igarashi et al., 2013). Two studies noted a decrease in *μ*/*α*–H*γ* and *β*–H*γ* PAC at movement onset, suggesting that these two types of PAC might suppress or “gate” movement (Miller et al., 2012; Yanagisawa et al., 2012). One study also found L*γ*–spike coupling (Igarashi et al., 2013), suggesting that there might also be coupling between L*γ* and H*γ*. Moreover, PAC has been investigated as a biomarker for closed-loop deep brain stimulation in treatment of movement disorders (De Hemptinne et al., 2015; Swann et al., 2015; Habets et al., 2018; Bouthour et al., 2019).

Here, we describe a novel form of PAC between L*γ* and H*γ* across species and motor behaviors of varying complexity. We recorded LFPs in monkeys during a reaching task and humans during finger-flexion and word-reading tasks. For each task, we computed L*γ*–H*γ* PAC using two different methods: the modulation index (MI) (Tort et al., 2010) and a generalized linear model (GLM) framework (Nadalin et al., 2019). We found L*γ*–H*γ* PAC in many parts of the motor and premotor cortices of monkeys and humans and showed that it modulates during these motor behaviors. To our knowledge, this is the first study to investigate L*γ*–H*γ* PAC. This finding provides new insight into the roles of different gamma band activities in motor and premotor cortices. Additionally, L*γ*–H*γ* PAC could potentially serve as a biomarker for studies of motor control or movement disorders.

## Materials and Methods

All experimental protocols were performed with approval from the Institutional Animal Care Use Committee and the Institutional Review Board of Northwestern University. All analyses were performed using custom scripts in MATLAB (MathWorks).

### Reaching task subjects and data acquisition

The monkey experimental protocols and results are reported in detail elsewhere (Flint et al., 2012). To summarize, two rhesus monkeys (C and M) were trained to perform a center-out reaching task while grasping a two-link planar manipulandum. The center-out reaching task involved moving a computer cursor via the manipulandum to one of eight square, 2 cm outer targets spaced at 45° intervals around a circle of radius 10 cm. Each trial began with the monkey holding the cursor in the center target of the circle. After a random hold time of 0.5–0.6 s, a randomly selected outer target illuminated, signaling the monkey to reach to that target. The monkey needed to move the cursor into the outer target within 1.5 s and hold for a random time of 0.2–0.4 s to receive a liquid reward.

An intracortical 96-channel silicon microelectrode array (Blackrock Neurotech) was implanted in the proximal arm area of M1 contralateral to the tested arm in monkeys C and M. Another intracortical 96-channel array was previously implanted in the proximal arm area of S1 contralateral to the tested arm in monkey M. Intracortical arrays were grounded to the Cereport pedestal and referenced to a subdural platinum wire with 3 mm exposed length placed under the dura. Anesthesia and surgery details are described elsewhere (Pohlmeyer et al., 2007; Flint et al., 2012).

Neural signals were recorded using a 128-channel acquisition system (Cerebus, Blackrock Neurotech) while the monkeys performed the reaching task. LFPs were obtained by bandpass filtering between 0.5 and 500 Hz and sampling at 2 kHz for monkey C and 1 kHz for monkey M, with subsequent notch filtering at 60, 120, 180, and 240 Hz to remove line noise. Multiple data files of 5–20 min duration were recorded in each 60–90-min-long experimental session. Overall, we analyzed 32 data files over 10 experimental sessions from C M1, 58 data files recorded over 11 sessions from M M1, and 48 data files recorded over 10 sessions from M S1. Movement onset was detected from synchronized kinematics recorded from the manipulandum as described elsewhere (Pohlmeyer et al., 2007; Flint et al., 2012).

### Finger-flexion task and data acquisition

All human participants were recruited at Northwestern Memorial Hospital and gave informed consent prior to participation. The experimental protocols and results are reported in detail elsewhere (Flint et al., 2020). Briefly, we analyzed recordings from five male human participants, four (FF1–FF4) undergoing awake intraoperative mapping prior to resection of low-grade gliomas and one (FF5) undergoing extraoperative intracranial monitoring before resection for medically refractory epilepsy.

Participants were instructed to execute repeated trials of a finger-flexion task that required isotonic movement and isometric force of a single finger in sequence. At the beginning of each trial, participants held their index finger in a neutral posture. After a cue on a monitor, they executed a flexion movement, bringing the palmar surface of the distal phalanx of the index finger into contact with a load cell. They then applied force to match a randomly generated force target presented on the monitor within 2 s. Following a successful match or failure, the participant returned the finger to the neutral position. The next trial began after a delay of 1 s. Target presentation and cursor feedback were conducted by the open-source BCI2000 software (Schalk et al., 2004). Finger kinematics were recorded with a 22-sensor CyberGlove (Immersion), sampled at 2 kHz. The time resolution for both kinematic data acquisition and force cursor control was 50 ms.

In FF1-4, ECoG arrays were placed over hand motor areas contralateral to the tested hand, which were defined using anatomical landmarks (i.e., “hand knob” in the precentral gyrus), preoperative fMRI, and/or direct electrocortical stimulation mapping to identify functional hand motor area. In FF5, arrays were placed according to clinical necessity. All participants had arrays covering M1 and premotor cortex, with all except for FF3 covering S1 as well. For FF1–FF4, 64-electrode (8 × 8) higher-density arrays (Integra), with 1.5 mm exposed recording site diameter and 4 mm interelectrode spacing, were used. For FF5, a 32-electrode (8 × 4) array, with the same electrode size and spacing as the 64-electrode arrays, was used. ECoG signals were bandpass filtered from 0.3 to 500 Hz and sampled at 2 kHz, and force and kinematics were synchronously recorded, using a NeuroPort Neural Signal Processor (Blackrock Microsystems).

### Word-reading task and data acquisition

The experimental protocols and results are reported in detail elsewhere (Mugler et al., 2014, 2018). Briefly, we analyzed data from seven human participants, six (WR1–WR6) during awake intraoperative mapping for glioma resection and one (WR7) during extraoperative intracranial monitoring for medically refractory epilepsy. A monitor presented randomly selected, single words either every 2 s (WR1–6) or every 4 s (WR7). Participants read the word aloud as soon as it appeared. The displayed word was randomly selected from a set of monosyllabic words with primarily consonant–vowel–consonant structure. This set consisted mostly of words from the modified rhyme test [details elsewhere (House et al., 1965)], as well as several additional words containing American English phonemes not seen in the modified rhyme test. Stimuli were presented using BCI2000 (Schalk et al., 2004). Speech audio was sampled at either 48 kHz from a unidirectional lapel microphone (Sennheiser) placed near the participant's mouth connected to a recording computer (WR1–WR6) or at 44.1 kHz from a USB microphone (MXL) using a customized version of BCI2000 and a Tucker-Davis Bioamp system (WR7).

ECoG arrays were placed over areas related to motor speech production, namely, ventral M1, ventral premotor cortex, and frontal operculum (inferior frontal gyrus). All electrode arrays except for WR6 covered portions of ventral S1 as well. Array location was confirmed as described above. Recordings in WR1–WR6 used 64-electrode, higher-density arrays and were recorded using the methods described above. Recordings in WR7 used a 32-electrode (8 × 4) clinical array (PMT), with 2.3 mm exposed diameter and 10 mm interelectrode spacing, and were recorded with a Nihon Kohden system, bandpass filtering from 0.5 to 300 Hz, and sampling at 1 kHz. Audio recordings were synchronized to the ECoG recordings.

### ECoG electrode localization

For intraoperative recordings, electrode locations were stereotactically registered at the time of grid placement using Brainlab Curve. We identified each electrode's functional anatomical position with regard to surrounding landmarks (i.e., central sulcus, precentral gyrus, frontal gyri) using the superposed electrode locations on the reconstructed cortical surface provided in the Brainlab software suite, as well as intraoperative photos. For extraoperative recordings, we used the Fieldtrip toolbox (Oostenveld et al., 2011) to reconstruct the patients’ cortical surface from the preimplantation MRI and coregistered it to the postimplantation CT scan. We verified our presumed electrode functional anatomical locations in both settings to be coherent with cortical stimulation mapping results. For ensemble visualization of electrodes from multiple participants, we translated our identified electrode positions to a template brain (Lalys et al., 2010) using LeGUI software (Davis et al., 2021).

### Signal processing

All analyses were performed using custom MATLAB (MathWorks) scripts unless otherwise specified. All intracortical and ECoG signals were resampled to 1 kHz and notch filtered at 60, 120, and 180 Hz to remove line noise. Afterward, each electrode was visually inspected for noise or artifacts and excluded from subsequent analyses if noisy. The clean channels in each ECoG array were common average referenced (CAR).

Trials were aligned to event onsets of each task. For the reaching task, changes in the 2D cursor position were used to identify reach (i.e., movement) onset (see Flint et al., 2012 for details). For the finger-flexion task, principal component analysis was performed on the finger joint positions measured by the CyberGlove sensors. The dominant component reflected the position of the index finger, and the derivative of this component was used to identify movement onset (see Flint et al., 2017 for details). For the word-reading task, visual and auditory spectral changes in the audio signal were inspected to manually label the onset of each phoneme within each word. Speech onset was identified as the onset of the first phoneme in each word.

LFP and ECoG spectrograms were computed in a 2 s interval centered on event onset using 256 ms bins of data, shifted in 25 ms increments. For each bin, a Hanning window and fast Fourier transform were applied, and the resulting complex magnitudes were squared. Spectrograms were created by computing the log of the mean magnitude over trials for each bin and normalizing by subtracting the log of the mean power spectrum over the entire interval. For power spectra, the aperiodic component was estimated using an iterative method (Donoghue et al., 2020).

### Estimation of phase–amplitude coupling

Phase–amplitude coupling (PAC) between the phase of the lower frequency band and the amplitude of the higher frequency band was computed using two methods: the modulation index (MI) (Tort et al., 2010) and a modified GLM framework (Nadalin et al., 2019). We selected the second method because it considers the power of the frequency-band–defining phase when estimating PAC, reducing the impact of an important confound and thus permitting a more valid interpretation of PAC changes with behavior (Aru et al., 2015; Nadalin et al., 2019). For each method, the CAR signals were first bandpass filtered with a two-way least-squares FIR filter using EEGLAB's *eegfilt.m* (Delorme and Makeig, 2004) to isolate activity within the L*γ* (40–50 Hz) and H*γ* (70–200 Hz) bands. We deliberately chose 40–50 Hz to represent L*γ* to avoid any potential overlap with *β* and with line noise. Each band's filtered signal was then *z*-scored in time. The Hilbert transform was then applied to these signals to extract the instantaneous L*γ* phase $\left(\phi_{L\gamma }\right)$ and H*γ* amplitude $\left(A_{H\gamma }\right)$, as well as the instantaneous L*γ* amplitude $\left(A_{L\gamma }\right)$ for the modified GLM framework. Using the event-onset times, the samples corresponding to the baseline and event-onset (reach, flexion, or voice) intervals from each trial were identified. Baseline and event-onset intervals were −500 to −300 ms and −100 to 100 ms, respectively, in monkeys and −600 to −400 ms and −200 to 0 ms, respectively, in humans. We chose slightly earlier intervals in humans because these recordings included premotor areas (anterior part of the precentral gyrus and anterior to the precentral sulcus), which activate earlier than M1 and S1 for a given movement.

To estimate PAC for each interval using the MI (Tort et al., 2010), the corresponding 200 ms bins of $\phi_{L\gamma }$ and $A_{H\gamma }$ from each trial were concatenated and sorted to create a histogram of amplitudes as a function of phases (20 phase bins equally spaced from −π to π). The MI value was then computed from the Kullback–Leibler divergence between the amplitude distribution as a function of $\phi_{L\gamma }$ and a uniform distribution. We then randomly shuffled trial pairs of $\phi_{L\gamma }$ and $A_{H\gamma }$ 1,000 times to create a distribution of surrogate MI values. The *z*-scored MI $\left(MI_{z}\right)$ was then computed by comparing the observed MI value to the mean MI value of the surrogate distribution, specifically as follows:

$$MI_{z}=\frac{{MI_{\mathrm{observed}}-\bar{\mathrm{MI}}}_{\mathrm{surrogate}}}{\sigma_{\mathrm{surrogate}}},\;\;$$ (1)

where higher values of $MI_{z}$ suggest stronger PAC.

Comodulograms were created using the MI by first defining two sets of frequency bands, one for the phase frequencies and the other for the amplitude frequencies. The phase frequency bands were centered on frequencies ranging from 4 to 56 Hz in steps of 4 Hz and had fixed bandwidths of 4 Hz. The amplitude frequency bands were centered on frequencies ranging from 10 to 200 Hz in steps of 10 Hz and had variable bandwidths. Specifically, the bandwidths of the amplitude frequency bands were twice the center frequency of the corresponding phase band, as this ensured that the passband encompassed the sidebands created by the assumed phase frequency (Berman et al., 2012). For each pair of phase and amplitude frequency bands, $MI_{z}$ was then computed as previously described to create comodulograms during the baseline and onset intervals.

To estimate PAC for each interval using the modified GLM framework (Nadalin et al., 2019), we concatenated and used the corresponding 200 ms bins of $\phi_{L\gamma }$, $A_{H\gamma }$, and $A_{L\gamma }$ from each trial to create three GLMs. Each GLM used a gamma distribution to model the conditional distribution of the response variable— $A_{H\gamma }$—given the predictor variable, where the mean parameter of the gamma distribution was related to the predictor variable via a link function. The first GLM defined the link function as a linear combination of spline basis functions to approximate the predictor variable, $\phi_{L\gamma }$. The second GLM defined the link function as a linear function to approximate the predictor variable, $A_{L\gamma }$. The third GLM defined the link function as a linear combination of the first two GLMs’ link functions and two terms that approximated the interaction between two predictor variables, $\phi_{L\gamma }\;$and $A_{L\gamma }$.

For PAC, the second and third GLMs were used to create surfaces in the 3D space spanned by $\phi_{L\gamma }$, $A_{L\gamma }$, and $A_{H\gamma }$ (Nadalin et al., 2019). The surface created with the second GLM, $S_{A_{L\gamma }}$, represented $A_{H\gamma }$ as a function of only $A_{L\gamma }$ and was thus constant in the $\phi_{L\gamma }$ dimension. The surface created with the third GLM, $S_{A_{L\gamma }\;\phi_{L\gamma }}$, represented $A_{H\gamma }$ as a function of both $\phi_{L\gamma }$ and $A_{L\gamma }$. The method's measure of PAC, $R_{\mathrm{PAC}}$, was the maximum absolute fractional difference between these two surfaces, defined as follows:

$$\begin{array}{c} R_{\mathrm{PAC}}=\max\left[\left|\frac{1-S_{A_{L\gamma }}}{S_{A_{L\gamma }\;\phi_{L\gamma }}}\right|\right], \end{array}\;\;$$ (2)

where higher values of $R_{\mathrm{PAC}}$ indicated stronger PAC. Surrogate $R_{\mathrm{PAC}}$ values were created by randomly shuffling the trial pairs of $\phi_{L\gamma }$, $A_{L\gamma }$, and $A_{H\gamma }$ and using the resulting concatenated signals to create the three GLMs. This was done 1,000 times to create a surrogate distribution.

### Analysis of phase–amplitude coupling

To identify electrodes with significant PAC within the baseline and event-onset intervals using the MI, $MI_{z}$ values during each interval were converted to one-sided *p* values and corrected for the number of electrodes (false discovery rate correction; *α* = 0.05). To do the same using the modified GLM framework, we defined *p* values as the proportion of surrogate $R_{\mathrm{PAC}}$ values greater than the estimated $R_{\mathrm{PAC}}$ and corrected for the number of electrodes (false discovery rate correction; *α* = 0.05). If the proportion was 0, then *p* was set to 0.0005 (Nadalin et al., 2019). Only electrodes with significant PAC during either the baseline or event-onset intervals were included for further analysis. For the participants performing the finger-flexion task, each electrode was labeled as either a precentral gyrus (including M1 and part of premotor cortex), postcentral gyrus, or region anterior to the precentral sulcus electrode (including premotor and prefrontal cortices). For the participants performing the word-reading task, each electrode was labeled as either a precentral gyrus, postcentral gyrus, or posterior inferior frontal gyrus electrode.

For each interval during the reaching task, the degree of L*γ*–H*γ* PAC was determined by computing the proportion of electrodes with significant PAC per file. Differences in L*γ*–H*γ* PAC between the intervals were assessed by subtracting $MI_{z}$ and $R_{\mathrm{PAC}}$ during the event-onset interval from $MI_{z}$ and $R_{\mathrm{PAC}}$ during the baseline interval, respectively, for each included electrode across all files. For each defined brain region and each interval in human participants performing the finger-flexion or word-reading task, the degree of L*γ*–H*γ* PAC was determined by computing the proportion of electrodes with significant PAC per participant. Differences in L*γ*–H*γ* PAC between the intervals were assessed by subtracting $MI_{z}$ and $R_{\mathrm{PAC}}$ during the event-onset interval from $MI_{z}$ and $R_{\mathrm{PAC}}$ during the baseline interval, respectively, for each included electrode in a region.

### Statistics

Statistical analyses were performed in MATLAB. For PAC results from the reaching task (monkeys), two-tailed paired and unpaired *t* tests were used to assess within and between electrode differences in PAC strength, respectively. For PAC results from the finger-flexion and word-reading tasks (humans), one-tailed Wilcoxon signed rank tests were used to assess within electrode differences in PAC strength.

### Data and code accessibility

The code and datasets used during the current study are available from the corresponding author on reasonable request.

## Results

**Figure 1.**
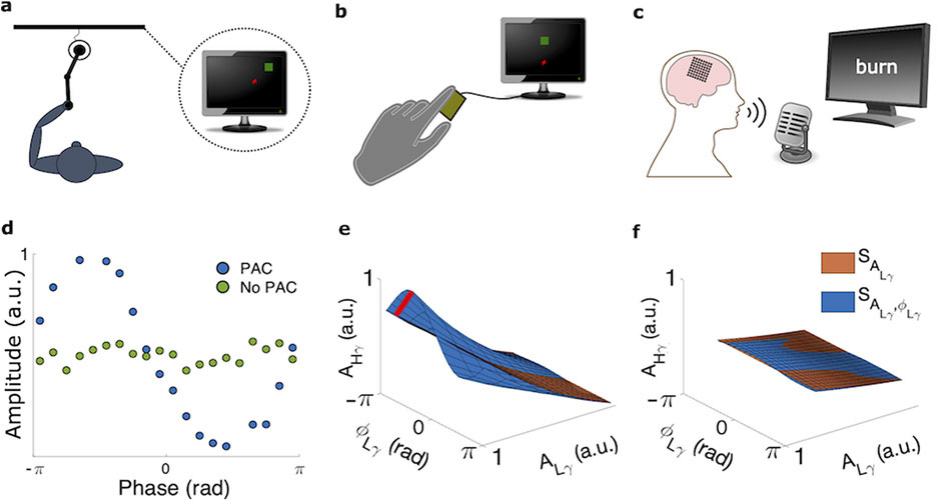
Motor behaviors and the phase–amplitude coupling (PAC) methods. ***a***, Monkey performing a reaching task using a planar manipulandum. ***b***, Human participant performing a finger-flexion task. The participant flexed the index finger and used isometric force to move a computer cursor in 1D to randomly placed target (see Materials and Methods). ***c***, Human participant reading single words from the screen (word-reading task). ***d–f***, Plots generated from an example electrode from the reaching task. ***d***, Example phase–amplitude plots of Hg amplitude at each Lg phase. Notable variation of the higher frequency amplitude with phase indicates PAC (blue), whereas little-to-no variation suggests no PAC (green). a.u., arbitrary units. ***e***, ***f***, Surfaces generated by the GLM framework demonstrating PAC (***e***) and no PAC (***f***). The orange surface ( $S_{A_{L\gamma }}$) depicts the higher frequency amplitude ( $A_{H\gamma }$) as a function of the lower frequency amplitude $\left(A_{L\gamma }\right)$. The blue surface is the $A_{H\gamma }$ as a function of both the lower frequency phase $\left(\phi_{L\gamma }\right)$ and $A_{L\gamma }$. The degree of PAC is directly proportional to the maximum orthogonal distance between the two surfaces (red line).

We collected intracranial recordings in monkeys and humans performing different motor behaviors (Fig. 1*a*,*c*). Two rhesus monkeys (C and M) performed a reaching task with visual feedback, during which we recorded neural activity from two intracortical arrays in the primary motor cortices (M1) of both monkeys (CM1 and MM1) and from one array in the primary somatosensory cortex (S1) of one (MS1) over multiple experimental sessions spanning 4–9 weeks (Fig. 1*a*). Furthermore, 12 human participants performed either a finger-flexion task (5 participants; Fig. 1*b*) or a word-reading task (7 participants; Fig. 1*c*; see Extended Data Table 1-1 for demographics). In these participants, we recorded neural activity from electrocorticography (ECoG) arrays covering the posterior frontal lobe and postcentral gyrus.

10.1523/ENEURO.0163-23.2023.t1-1 Table 1-1 Participant demographics. Download Table 1-1, DOCX file.

### L*γ*–H*γ* phase–amplitude coupling is a marker of rest and reaching in monkey M1

**Figure 2.**
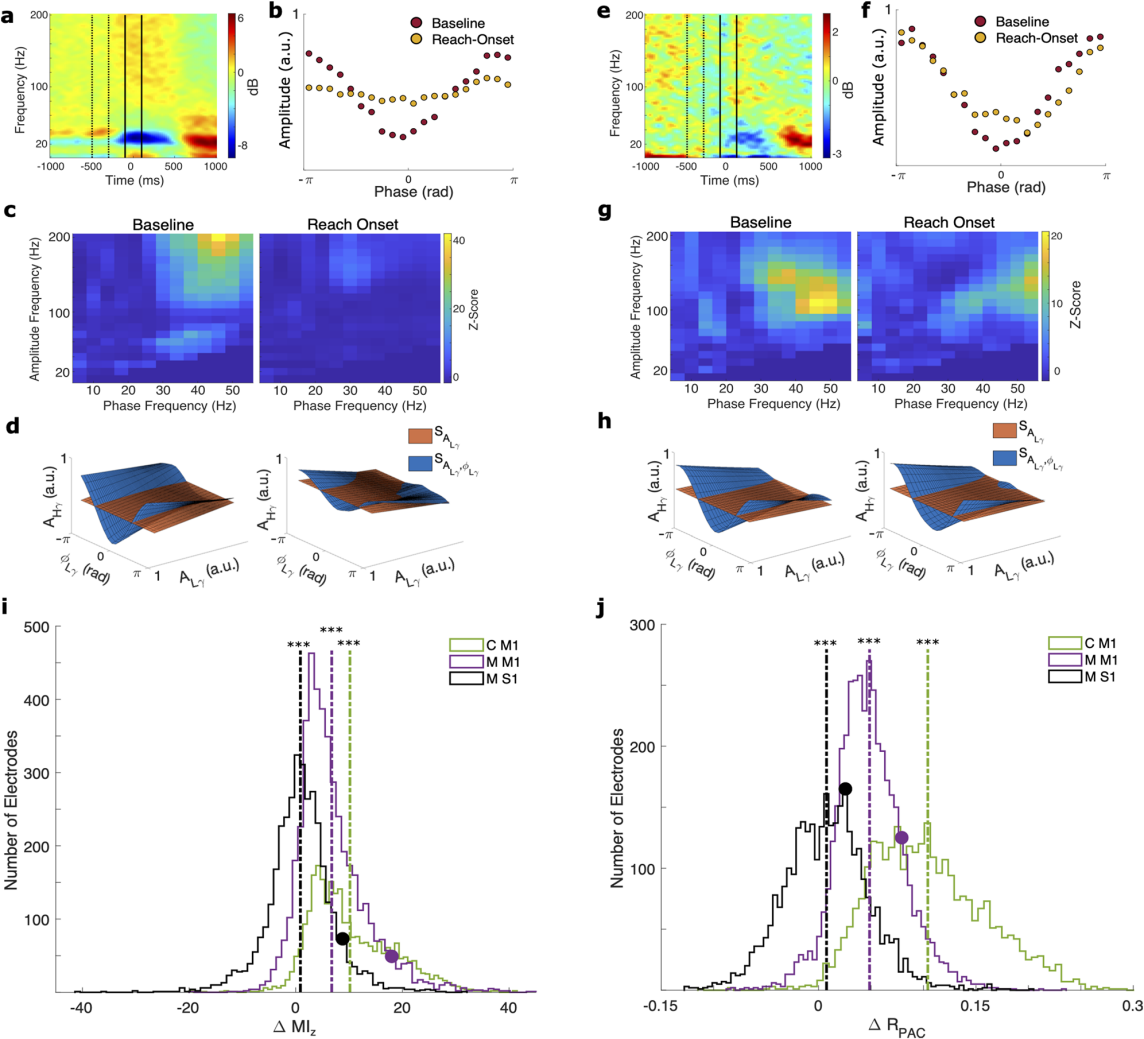
Low *γ*–high *γ* PAC in monkeys performing the reaching task. ***a–d***, Illustrative plots from an example electrode and experimental session in M1 in monkey M (M M1). ***a***, Spectrogram time-locked to reach onset with the baseline (−500 to −300 ms, dashed lines) and reach-onset (−100 to 100 ms, solid lines) intervals marked. ***b***, Phase–amplitude plots during the baseline (red) and reach-onset (yellow) intervals. ***c***, Comodulograms during the baseline (left) and reach-onset (right) intervals. ***d***, Surfaces generated by the GLM framework during the baseline (left) and reach-onset (right) intervals. The greater the differences between the surfaces, the greater the PAC. Thus, PAC decreases from baseline to reach onset. ***e–h***, Same as in ***a–d***, except in monkey M S1 (M S1). ***i***, Distributions of the differences in the *z*-scored modulation index $\left(\Delta MI_{z}\right)$ between intervals (baseline minus reach onset) in each electrode over all experimental sessions for M1 in monkey C (C M1), M M1, and M S1. Vertical dashed lines represent mean $\Delta MI_{z}$ for each monkey/region. Circles represent exemplary electrodes shown in ***a–d*** (purple) and ***e–h*** (black). $\Delta MI_{z}$ was significantly >0 in all three cases (***p < 0.001), with much higher means in M1 and M1 than S1. ***j***, Same as in ***i***, but for $\Delta R_{\mathrm{PAC}}$. $\Delta R_{\mathrm{PAC}}$ was significantly >0 in all three cases, with much higher means in M1 than in S1. See Extended Data Figure 2-1 for an example electrode demonstrating other types of PAC.

In the sensorimotor cortex, descriptions of *θ*–L*γ* (Igarashi et al., 2013), *μ*/*α*–H*γ* (Yanagisawa et al., 2012), and *β*–H*γ* (Miller et al., 2012; De Hemptinne et al., 2013) PAC and their modulation by movement (Miller et al., 2012; Yanagisawa et al., 2012) have provided insight into the temporal gating of motor representation in the sensorimotor cortex during movement execution. To add to these previous findings, we investigated the existence of L*γ*–H*γ* PAC in the sensorimotor cortex of monkeys and whether it modulates with movement. For each experimental session during the reaching task, we aligned the trials to the outward reach onset, seeing typical modulation of H*γ* power around reach onset in precentral and postcentral gyri (Fig. 2*a*,*b*). We defined two intervals: resting baseline in the center target (−500 to −300 ms) and reach onset (−100 to 100 ms). For each electrode and interval, we estimated the L*γ*–H*γ* PAC using the *z*-scored modulation index ( $MI_{z}$; Fig. 1*d*; Tort et al., 2010) and a GLM framework measure ( $R_{\mathrm{PAC}}$; Fig. 1*e*; Nadalin et al., 2019). We only included electrodes demonstrating significant L*γ*–H*γ* PAC identified during either interval for statistical comparisons.

10.1523/ENEURO.0163-23.2023.f2-1 Figure 2-1 Comodulogram of example electrode demonstrating other types of phase-amplitude coupling (β-Hγ PAC here) in addition to Lγ-Hγ PAC during the reaching task. Download Figure 2-1, TIF file.

We observed a high degree of L*γ*–H*γ* PAC in all three intracortical arrays using both $MI_{z}$ and $R_{\mathrm{PAC}}$ (Fig. 2). Using $MI_{z}$ and $R_{\mathrm{PAC}}$, most electrodes demonstrated significant L*γ*–H*γ* PAC during either interval for CM1, MM1, and MS1 (Table 1). For some electrodes in M1 and S1, comodulograms created using $MI_{z}$ demonstrated that L*γ*–H*γ* PAC was the predominant type of PAC (based on visual inspection), especially during the baseline interval (Fig. 2*c*,*g*). This was not a consistent observation, as other types of PAC previously reported in the sensorimotor cortex, such as *β*–H*γ* PAC (Miller et al., 2012; De Hemptinne et al., 2015), were predominant in other electrodes (Extended Data Fig. 1-1).

**Table 1.** Summary of results from the reaching task

Location	Total number of electrodes	Significant electrodes^a^		Mean ΔPAC^b^	
		$MI_{z}$	$R_{\mathrm{PAC}}$	$\Delta MI_{z}$	$\Delta R_{\mathrm{PAC}}$
CM1	2,942	2,728 (92.7%)	2,899 (98.5%)	10.16	0.10
MM1	5,435	4,548 (83.7%)	4,188 (77.1%)	6.74	0.049
MS1	4,351	3,633 (83.5%)	3,194 (73.4%)	0.87	0.0076

^a^ Number and proportion of electrodes with significant phase–amplitude coupling (PAC), measured using the *z*-scored modulation index $\left(MI_{z}\right)$ or the GLM framework $\left(R_{\mathrm{PAC}}\right)$ during either the baseline or reach-onset interval.

^b^ The mean change (Δ) in PAC ( $MI_{z}$ or $R_{\mathrm{PAC}}$) per electrode between the baseline and reach-onset intervals (i.e., baseline minus reach onset). CM1, M1 of monkey C; MM1, M1 of monkey M; MS1, S1 of monkey M.

We also found a differential modulation of L*γ*–H*γ* PAC with reaching based on brain region (Table 1). Across all electrodes with significant L*γ*–H*γ* PAC identified with $MI_{z}$, $MI_{z}$ was significantly higher during baseline than during reach onset in CM1 (two-tailed paired *t* tests; *p* < 0.0001) and MM1 (*p* < 0.0001), and this pattern was seen in most electrodes in CM1 and MM1 (Fig. 2*i*). Likewise, across all electrodes with significant L*γ*–H*γ* PAC identified with $R_{\mathrm{PAC}}$, the baseline $R_{\mathrm{PAC}}$ was significantly higher than the reach onset $R_{\mathrm{PAC}}$ in CM1 (*p* < 0.0001) and MM1 (*p* < 0.0001), and this pattern was seen in nearly all electrodes in CM1 and MM1 (Fig. 2*j*).

For MS1, we also observed significantly higher L*γ*–H*γ* PAC in baseline than in reach onset using both $MI_{z}$ (two-tailed paired *t* tests; *p* < 0.0001) and $R_{\mathrm{PAC}}$ (*p* < 0.0001; Table 1). However, the mean within-electrode difference in $MI_{z}$ and $R_{\mathrm{PAC}}$ between the two intervals (i.e., baseline minus reach onset) across all electrodes with significant L*γ*–H*γ* PAC was much smaller in MS1 than that in MM1 and CM1 (Table 1; dashed lines in Fig. 2*i*,*j*). Compared with the overall distribution of differences in $MI_{z}$ in MS1, the distributions of differences in $MI_{z}$ were significantly greater in both MM1 (two-tailed unpaired *t* tests; *p* < 0.0001) and CM1 (*p* < 0.0001). Likewise, the distributions of differences in $R_{\mathrm{PAC}}$ in MM1 (*p* < 0.0001) and CM1 (*p* < 0.0001) were significantly greater than the $R_{\mathrm{PAC}}$ distribution in MS1. Indeed, a relatively smaller proportion of MS1 electrodes had a greater baseline than reach-onset $MI_{z}$ (57.3%) and $R_{\mathrm{PAC}}$ (58.0%) compared with MM1 ( $MI_{z}$, 91.4%; $R_{\mathrm{PAC}}$, 95.0%) and CM1 ( $MI_{z}$, 96.0%; $R_{\mathrm{PAC}}$, 99.2%). These results indicate a regional influence on degree of modulation of L*γ*–H*γ* PAC with movement.

### L*γ*–H*γ* phase–amplitude coupling is a marker of finger flexion versus rest in humans

Our initial results confirm the existence of L*γ*–H*γ* PAC in the sensorimotor cortex of monkeys and indicate a movement- and region-related modulation of L*γ*–H*γ* PAC. To support and expand upon these initial findings, we investigated L*γ*–H*γ* PAC in humans performing a finger-flexion task. Briefly, the participants were visually cued to flex their index finger and then extend back to a neutral baseline (see Materials and Methods). We categorized electrodes as precentral gyrus (preCG), postcentral gyrus (postCG), or anterior to the precentral sulcus (aPreCS, including premotor and prefrontal cortices) electrodes depending on their estimated location. We defined baseline (−600 to −400 ms) and flexion-onset (−200 to 0 ms) intervals relative to the onset of finger flexion using slightly earlier times than for the monkeys because there were multiple electrodes in premotor cortex (which activates sooner) included in the analysis. We computed $MI_{z}$ and $R_{\mathrm{PAC}}$ for each interval and electrode, pooled results over all participants based on their respective interval and electrode category, and only included electrodes demonstrating significant L*γ*–H*γ* PAC during either interval for statistical comparisons.

**Figure 3.**
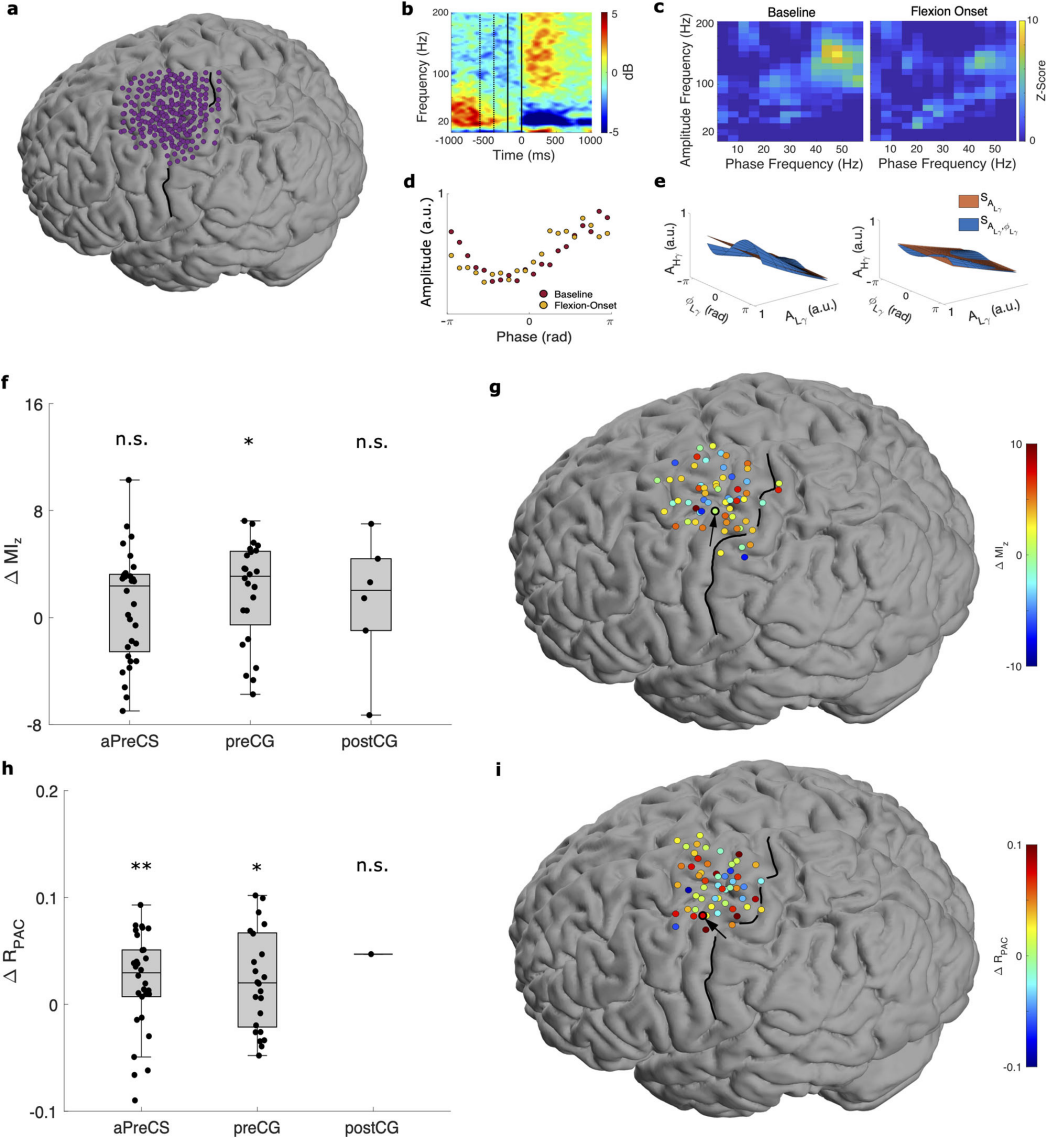
Low *γ*–high *γ* PAC in humans performing finger flexion. ***a***, Spatial distribution of all electrodes across all five participants plotted on a template brain. ***b–d***, Illustrative plots from an example electrode in motor cortex (circled in black/arrow in ***g***). ***b***, Spectrogram time-locked to flexion onset (top, left) with baseline (−600 to −400 ms, dashed lines) and flexion-onset (−200 to 0 ms, solid lines) intervals marked. **c**, Comodulograms during the baseline (left) and flexion-onset (right) intervals. See Extended Data Figure 3-1 for an example electrode demonstrating other types of PAC. ***d***, Phase–amplitude plots during the baseline (red) and flexion-onset (yellow) intervals. ***e***, Surfaces generated by the GLM framework during the baseline (left) and flexion-onset (right) intervals from another example electrode (circled in black/arrow in ***i***). ***f***, Differences in $MI_{z}$ between intervals (baseline minus flexion onset) per electrode over all participants. $\Delta MI_{z}$ was significantly >0 for precentral gyrus (preCG) electrodes (*p < 0.05), but not for anterior to the precentral sulcus (aPreCS) and postcentral gyrus (postCG) electrodes (n.s.). ***g***, Spatial distribution of $\Delta MI_{z}$ for significant electrodes with the example electrode marked (black). ***h***, Same as in ***c***, except for $\Delta R_{\mathrm{PAC}}$. $\Delta R_{\mathrm{PAC}}$ was significantly >0 for aPreCS (**p < 0.01) and preCG electrodes (*p < 0.05), but not for postCG electrodes (n.s.). ***i***, Same as in ***g***, except for $\Delta R_{\mathrm{PAC}}$.

We found L*γ*–H*γ* PAC in all three defined brain regions using both $MI_{z}$ and $R_{\mathrm{PAC}}$ (Fig. 3). Using $MI_{z}$, we identified many preCG, postCG, and aPreCS electrodes with significant L*γ*–H*γ* PAC during either interval (Table 2). Of these, most preCG, postCG, and aPreCS electrodes had a greater $MI_{z}$ at baseline than flexion onset (Fig. 3*f*,*g*). Additionally, L*γ*–H*γ* PAC was the predominant or codominant type of PAC (based on visual inspection) in some electrodes but not all (Fig. 3*c* and Extended Data Fig. 2-1). Using $R_{\mathrm{PAC}}$, we identified several preCG, postCG, and aPreCS electrodes with significant L*γ*–H*γ* PAC during either interval (Table 2). Of these, most preCG, postCG, and aPreCS electrodes had a greater $R_{\mathrm{PAC}}$ at baseline than flexion onset (Fig. 3*h*,*i*).

10.1523/ENEURO.0163-23.2023.f3-1 Figure 3-1 Comodulogram of example electrode demonstrating other types of phase-amplitude coupling (θ-Hγ, μ/α-Lγ, μ/α-Hγ, and β-Hγ PAC here) in addition to Lγ-Hγ PAC during the finger-flexion task. Download Figure 3-1, TIF file.

**Table 2.** Summary of results from the finger-flexion task

Location	Total number of electrodes	Significant electrodes^a^		Median ΔPAC^b^	
		$MI_{z}$	$R_{\mathrm{PAC}}$	$\Delta MI_{z}$	$\Delta R_{\mathrm{PAC}}$
preCG	98	24 (24.5%)	25 (25.5%)	3.08	0.020
postCG	26	6 (23.1%)	1 (3.8%)	2.04	0.047
aPreCS	134	32 (23.9%)	30 (22.4%)	2.35	0.030

^a^ Number and proportion of electrodes with significant phase–amplitude coupling (PAC), measured using the *z*-scored modulation index $\left(MI_{z}\right)$ or the GLM framework $\left(R_{\mathrm{PAC}}\right)$ during either the baseline or flexion-onset interval.

^b^ The median change (Δ) in PAC ( $MI_{z}$ or $R_{\mathrm{PAC}}$) per electrode between the baseline and flexion-onset intervals (i.e., baseline minus flexion onset). preCG, precentral gyrus; postCG, postcentral gyrus; aPreCS, anterior to the precentral sulcus.

In monkeys, we showed that L*γ*–H*γ* PAC modulates with movement greatly in M1 and less so in S1 (Fig. 2*i*,*j*). One advantage of ECoG over intracortical arrays is much broader coverage, allowing us to investigate L*γ*–H*γ* PAC in more areas. We computed the difference in the pooled $MI_{z}$ and $R_{\mathrm{PAC}}$ (across patients and electrodes) between the two intervals (baseline minus flexion onset) per cortical region (Table 2). We found that in preCG, PAC significantly decreased moving from the baseline to flexion-onset interval using both the pooled $MI_{z}$ (one-tailed Wilcoxon signed rank test; *p* = 0.015) and $R_{\mathrm{PAC}}$ (*p* = 0.026). In contrast, in postCG, there was no change between baseline and flexion-onset intervals in either the pooled $MI_{z}$ (*p* = 0.28) or $R_{\mathrm{PAC}}$ (*p* = 0.50; Fig. 3*f*,*h*). Interestingly, we observed no change in the pooled aPreCS $MI_{z}$ (*p* = 0.139) but did find a significant decrease in the pooled aPreCS $R_{\mathrm{PAC}}$ (*p* = 7.27 × 10^−3^) moving from the baseline to flexion-onset interval (Fig. 3*f*,*h*). Although the $MI_{z}$ results showed only a nonsignificant trend, the significant decrease in $R_{\mathrm{PAC}}$ in aPreCS could indicate that the movement-related modulation of L*γ*–H*γ* PAC extends into the premotor/prefrontal region, as the GLM framework permits a more accurate interpretation of PAC (Nadalin et al., 2019).

### L*γ*–H*γ* phase–amplitude coupling discriminates between silence and speech onset in humans

**Figure 4.**
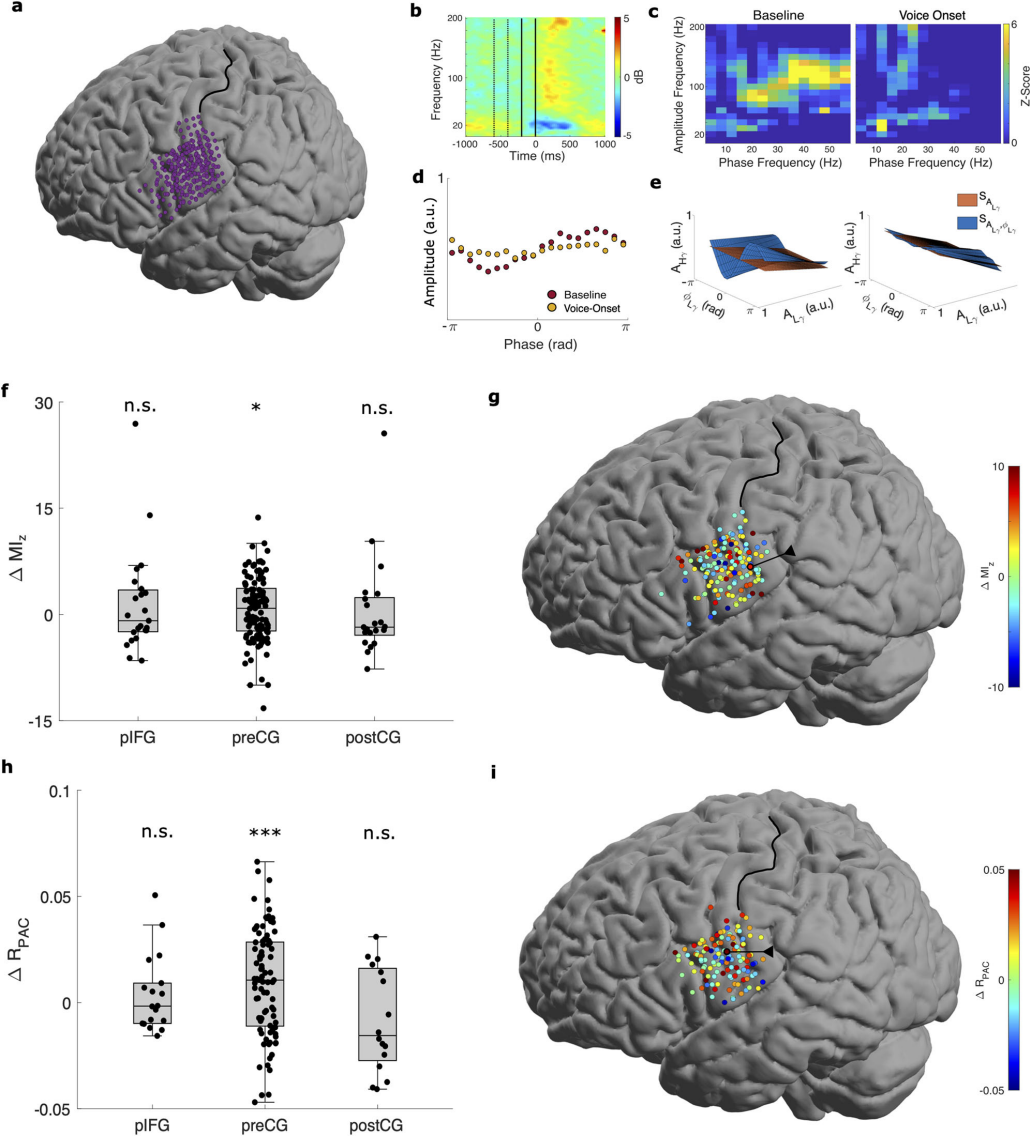
Low *γ*–high *γ* PAC in humans reading words. ***a***, Spatial distribution of all electrodes across all seven participants on a template brain. ***b–e***, Illustrative plots from an example electrode (circled in black in ***g***). ***b***, Spectrogram time-locked to voice onset (top, left) with baseline (−600 to −400 ms, dashed lines) and voice-onset (−200 to 0 ms, solid lines) intervals marked. ***c***, Comodulograms during the baseline (left) and voice-onset (right) intervals. See Extended Data Figure 4-1 for an example electrode demonstrating other types of PAC. See Extended Data Figure 4-2 for a more detailed comparison between *β*–H*γ* and L*γ*–H*γ* PAC in an example electrode. ***d***, Phase–amplitude plots during the baseline (red) and voice-onset (yellow) intervals. ***e***, Surfaces generated by the GLM framework during the baseline (left) and voice-onset (right) intervals from another example electrode (circled in black in ***i***). ***f***, Differences in $MI_{z}$ between intervals (baseline minus voice onset) per electrode over all participants. $\Delta MI_{z}$ was significantly >0 for preCG electrodes (*p < 0.05), but not for posterior inferior frontal gyrus (pIFG) and postCG electrodes (n.s.). ***g***, Spatial distribution of $\Delta MI_{z}$ for significant electrodes with the example electrode marked (circled in black/arrow). ***h***, Same as in ***c***, except for $\Delta R_{\mathrm{PAC}}$. $\Delta R_{\mathrm{PAC}}$ was significantly >0 for preCG electrodes (***), but not for pIFG (n.s.) and postCG electrodes (n.s.). ***i***, Same as in ***g***, except for $\Delta R_{\mathrm{PAC}}$ (example electrode circled in black).

Thus far, we have demonstrated that L*γ*–H*γ* PAC and its modulation patterns with simpler limb movements are consistently present and generalize across species. To examine whether these patterns were present in other types of movement, we investigated L*γ*–H*γ* PAC in humans performing a more complex motor behavior—speech. We categorized electrodes as preCG, postCG, or posterior inferior frontal gyrus (pIFG), depending on their estimated location, in participants who performed a word-reading task (Fig. 4*a*). As in the finger flexion participants, we computed $MI_{z}$ and $R_{\mathrm{PAC}}$ for each categorized electrode during the baseline (−600 to −400 ms) and voice-onset (−200 to 0 ms) intervals.

10.1523/ENEURO.0163-23.2023.f4-1 Figure 4-1 Comodulogram of example electrode demonstrating other types of phase-amplitude coupling (μ/α-Hγ, β-Lγ, and β-Hγ PAC here) in addition to Lγ-Hγ PAC during the word-reading task. Download Figure 4-1, TIF file.

10.1523/ENEURO.0163-23.2023.f4-2 Figure 4-2 Example comodulograms from the word-reading task during the baseline and event-onset (voice-onset) intervals. These demonstrate different modulation patterns of β-Hγ and Lγ-Hγ PAC, as measured by the mean z-scored modulation index () across the wide PAC bands encompassing both β (16-24 Hz, white) and Lγ (40-48 Hz, red) with Hγ (70-200 Hz). **a** Increase β-Hγ PAC from baseline ( $\bar{MI_{z}}$ of 0.87) to event-onset ( $\bar{MI_{z}}$ of 1.48) intervals despite decrease in Lγ-Hγ PAC from baseline ( $\bar{MI_{z}}$ of 6.03) to event-onset ( $\bar{MI_{z}}$ of 1.94) intervals. **b** D $\bar{MI_{z}}$ecrease in β-Hγ PAC from baseline ( $\bar{MI_{z}}$ of 2.06) to event-onset ( $\bar{MI_{z}}$ of -0.13) intervals despite increase in Lγ-Hγ PAC from baseline ( $\bar{MI_{z}}$ of 0.68) to event-onset ( $\bar{MI_{z}}$ of 1.94) intervals. Download Figure 4-2, TIF file.

We again found L*γ*–H*γ* PAC in all three speech-related brain regions using both $MI_{z}$ and $R_{\mathrm{PAC}}$ (Fig. 4). Using $MI_{z}$, we identified many preCG, postCG, and pIFG electrodes with significant L*γ*–H*γ* PAC during either interval (Table 3). Of these, most preCG and some postCG and pIFG electrodes had a greater baseline than voice-onset $MI_{z}$ (Fig. 4*f*,*g*). L*γ*–H*γ* PAC was the predominant or codominant type of PAC in some electrodes but not all (Fig. 4*c* and Extended Data Fig. 3-1). Similarly, we identified many preCG, postCG, and pIFG electrodes with significant L*γ*–H*γ* PAC during either interval using $R_{\mathrm{PAC}}$ (Table 3). Of these, most preCG and many postCG and pIFG electrodes had a greater baseline than voice-onset $R_{\mathrm{PAC}}$ (Fig. 4*h*,*i*).

**Table 3.** Summary of results from the word-reading task

Location	Total number of electrodes	Significant electrodes^a^		Median ΔPAC^b^	
		$MI_{z}$	$R_{\mathrm{PAC}}$	$\Delta MI_{z}$	$\Delta R_{\mathrm{PAC}}$
preCG	172	109 (63.4%)	89 (51.7%)	0.88	0.011
postCG	44	21 (47.7%)	16 (36.4%)	−0.87	−0.0017
pIFG	48	23 (47.9%)	18 (37.5%)	−1.78	−0.016

^a^ Number and proportion of electrodes with significant phase–amplitude coupling (PAC), measured using the *z*-scored modulation index $\left(MI_{z}\right)$ or the GLM framework $\left(R_{\mathrm{PAC}}\right)$ during either the baseline or voice-onset interval.

^b^ The mean change (Δ) in PAC ( $MI_{z}$ or $R_{\mathrm{PAC}}$) per electrode between the baseline and voice-onset intervals (i.e., baseline minus voice onset). preCG, precentral gyrus; postCG, postcentral gyrus; pIFG, posterior inferior frontal gyrus.

As we found for monkeys and humans doing finger flexion, we found region-related modulation of L*γ*–H*γ* PAC around word vocalization (Fig. 4*f–i*). The pooled preCG $MI_{z}$ (Wilcoxon signed rank test; *p* = 0.048) and *R*_PAC_ (*p* = 6.2 × 10^−4^) significantly decreased moving from the baseline to voice-onset interval (Table 3). In contrast, the pooled $MI_{z}$ and *R*_PAC_ in the pIFG ( $\Delta MI_{z}$ *p* = 0.36; $\Delta R_{\mathrm{PAC}}$ *p* = 0.43) and postCG ( $\Delta MI_{z}$ *p* = 0.77; $\Delta R_{\mathrm{PAC}}$ *p* = 0.91) did not change significantly between the two intervals (Table 3; Fig. 4*f*,*h*).

## Discussion

Generating movement and speech requires the coordination and control of neurons within brain motor and speech networks. Here, we examined cortical recordings in monkeys and humans for L*γ*–H*γ* PAC during and before movement and speech. We confirmed that L*γ*–H*γ* PAC is widespread across different motor regions, behaviors, and species. Furthermore, we observed a consistent, region-related modulation of L*γ*–H*γ* PAC during these motor behaviors across species. We found that L*γ*–H*γ* PAC was high in resting states and decreased at the onset of movement in both monkeys and humans in primary motor cortex. These modulations were independent of L*γ* amplitude modulations. This PAC was much less prevalent and remained relatively unchanged at movement onset, in postcentral gyrus in both species. Further, we observed similar, though less consistent, decreases in L*γ*–H*γ* PAC in higher-order motor regions of humans at the onset of movement. Moreover, these patterns were consistent across all three motor tasks. Collectively, these results suggest that modulation of Lγ–H*γ* PAC is a motor-related phenomenon that reflects underlying network dynamics fundamental to the gating and activation of motor behaviors.

Event-related modulation of L*γ* and H*γ* activity has been observed in many brain regions, in several species, and during both motor and nonmotor behaviors (Crone et al., 1998, 2001a,b, 2006; Jia and Kohn, 2011). Although sometimes combined in analyses, L*γ* and H*γ* are distinct entities associated with different origins and neural processes (Crone et al., 1998; Edwards et al., 2005; Canolty et al., 2006; Ray et al., 2008; Jia and Kohn, 2011; Ray and Maunsell, 2011; Buzsáki et al., 2012; Igarashi et al., 2013). L*γ* activity is thought to arise from rhythmic interactions between reciprocally connected inhibitory interneurons and excitatory pyramidal neurons (Buzsáki and Wang, 2012). In contrast, multiple studies have shown that H*γ* activity is a broadband (nonoscillatory) phenomenon likely arising from summed postsynaptic potentials of many thousands of neurons (Manning et al., 2009; Buzsáki et al., 2012; Miller et al., 2012; Donoghue et al., 2020). H*γ* is somewhat correlated with ensemble spiking activity (Ray et al., 2008; Jia and Kohn, 2011; Ray and Maunsell, 2011). Functionally, observations of spike–L*γ* phase coupling (Fries et al., 2001; Pesaran et al., 2002; Womelsdorf et al., 2006, 2007; Canolty et al., 2010; Igarashi et al., 2013) and synchronization of L*γ* phase across brain areas led to the communication through coherence (CTC) hypothesis (Fries, 2009, 2015), which posits that L*γ* band has a mechanistic role in neural communication by helping to synchronize across brain areas. Although the ability for L*γ* activity to directly influence neural activity is controversial (Fröhlich and McCormick, 2010; Engelhard et al., 2013; Buzsáki and Schomburg, 2015; Schneider et al., 2021), it appears clearer that *γ* activity, especially in the L*γ* range, is at least a marker of engaged, cross-area neural networks (Jia and Kohn, 2011; Engelhard et al., 2013). For example, L*γ* activity may coordinate spiking between hippocampus and rhinal cortices (Bauer et al., 2007), consistent with the observation that increasing L*γ* activity via biofeedback correlates with increased spiking synchronization (Engelhard et al., 2013). It also plays a strong role in spatial memory consolidation, as shown by causal closed-loop control of L*γ* (Kanta et al., 2019). Moreover, in an Alzheimer’s disease mice model, optogenetic L*γ* stimulation restored previously diminished L*γ* activity and improved spatial memory (Etter et al., 2019).

L*γ* synchronization (CTC) and PAC are both thought to be indicative of information transfer in a cortical network (Fries, 2009, 2015; Canolty and Knight, 2010). Moreover, L*γ* synchronization and PAC are related and may interact with each other (Gonzalez et al., 2020). One seminal study reported coupling between *θ* phase and H*γ* amplitude (*θ*–H*γ*) over a range of sensorimotor and cognitive tasks across the human cortex (Canolty et al., 2006). Additionally, several studies have observed *θ*–L*γ* and *θ*–H*γ* PAC in rat M1 (Igarashi et al., 2013), *μ*/*α*–H*γ* PAC in human sensorimotor cortices (Yanagisawa et al., 2012), and *β*–H*γ* PAC in human sensorimotor cortices (Miller et al., 2012; De Hemptinne et al., 2013) during upper extremity movements. Yet, to our knowledge, this study is the first to extensively investigate and report the presence of interactions between L*γ* and H*γ* activity via PAC. While we cannot definitively assign a mechanistic role to L*γ*–H*γ* PAC due to limitations of PAC analysis (Aru et al., 2015), one interpretation of our results is that this phenomenon is a signature of a fundamental neural process that suppresses motor-related activity on a more local scale. This is similar to reports that *μ*/*α*–H*γ* (Yanagisawa et al., 2012) and *β*–H*γ* (Miller et al., 2012) PAC decrease with movement, suggesting an inverse relationship with (sometimes called gating of) motor activity. Accordingly, local release from this suppressive process occurs in areas important for generating the desired movement—such as regions of M1 projecting to agonist muscles—which is reflected by a decrease in L*γ*–H*γ* PAC in electrodes recording from these areas. On a larger spatial scale, a more global reduction in this suppressive process, reflected by a net decrease in L*γ*–H*γ* PAC over a region, permits the transition from an inactive to active motor state.

What is the neural process that gives rise to L*γ*–H*γ* PAC? Since H*γ* activity has been hypothesized to be a marker of ensemble spiking activity (Ray et al., 2008; Jia and Kohn, 2011; Ray and Maunsell, 2011), one possibility is that L*γ*–H*γ* PAC is the LFP representation of spike–L*γ* correlative metrics, such as spike–L*γ* coherence. This would relate L*γ*–H*γ* PAC to the theorized functions of L*γ* activity (Fries, 2009, 2015). Indeed, in M1 of rats performing forelimb movements, spiking activity in shallow cortical layers preferentially occurred at specific L*γ* phases (Igarashi et al., 2013). Since multiple animal studies have investigated spike–L*γ* correlations (Fries et al., 2001; Womelsdorf et al., 2006, 2007; Engelhard et al., 2013; Igarashi et al., 2013; Zhou et al., 2016), especially in a sensory context, it would be interesting to see if L*γ*–H*γ* PAC is also present in similar scenarios to support this possibility. If so, L*γ*–H*γ* PAC as a surrogate for spike–L*γ* correlative metrics could be a useful investigative tool, especially in humans. Spiking information is difficult to obtain in this population, and surface electrode arrays and depth electrodes provide opportunities to record neural activity across large spatial scales. Alternatively, H*γ* has been shown to be correlated with underlying latent spiking dynamics (Gallego-Carracedo et al., 2022). It remains to be seen how L*γ*–H*γ* PAC may relate to the latent spiking dynamics.

**Figure 5.**
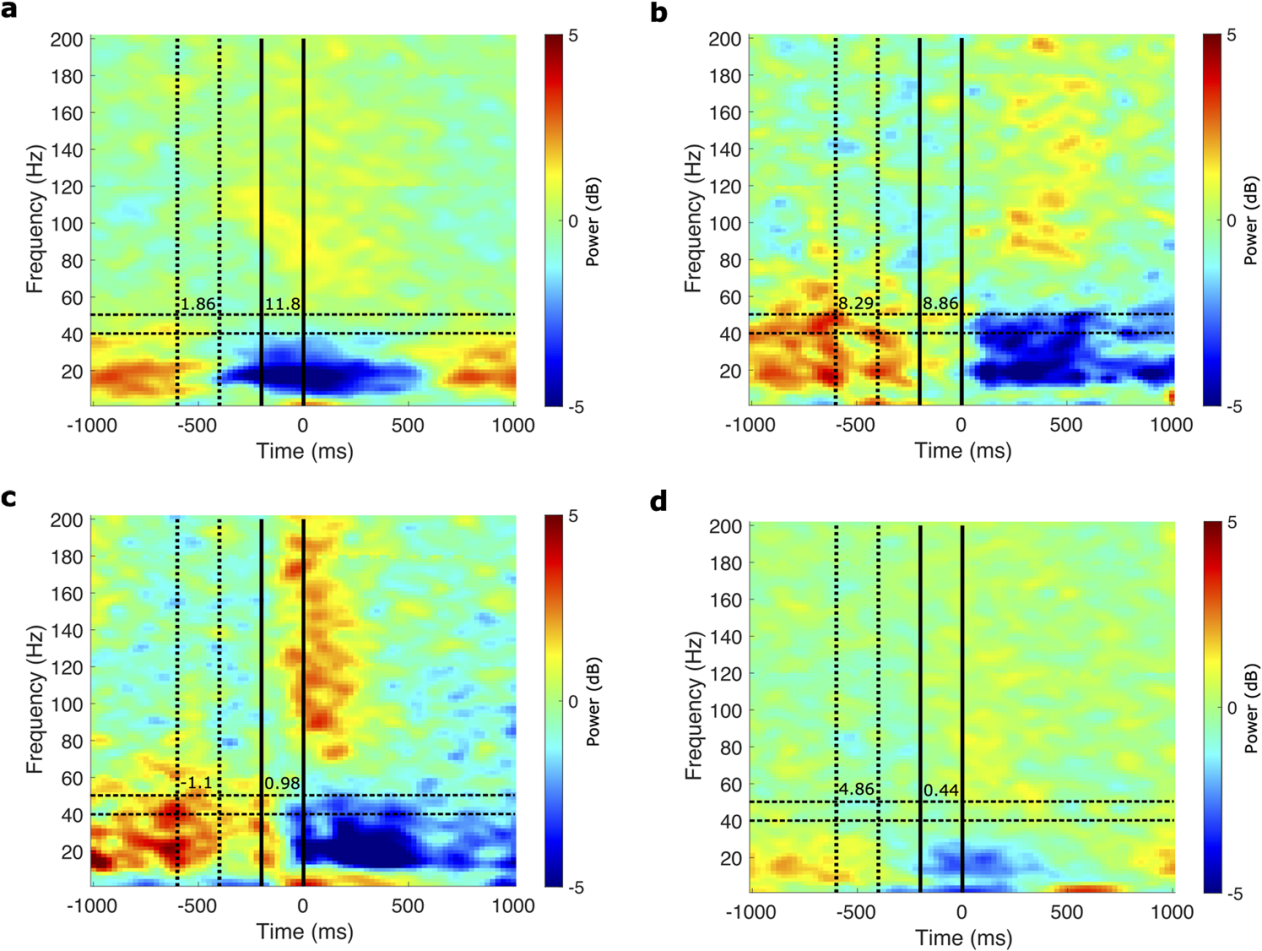
Spectrograms of example ECoG electrodes during the finger-flexion and word-reading tasks. ***a***, Decrease in L*γ* (40–50 Hz, horizontal dashed black lines) power (by 0.80 dB) from the baseline (vertical dotted black lines) to motor-onset (vertical solid black lines) intervals in an electrode with significant L*γ*–H*γ* PAC. PAC was measured by the *z*-scored modulation index $\left(MI_{z}\right)$, during both intervals (specified by the numbers between the lines for each interval). The $\Delta MI_{z}$ between the two intervals (baseline minus motor onset) was −9.94, indicating an increase in L*γ*–H*γ* PAC with motor onset. ***b***, Decrease in L*γ* power from the baseline to motor-onset intervals in an electrode with significant $MI_{z}$ during both intervals. $\Delta MI_{z}$ was −0.58, suggesting little to no change in L*γ*–H*γ* PAC with motor onset. ***c***, An electrode with no significant $MI_{z}$ during either interval despite relatively high L*γ* power during baseline. ***d***, Small to no change in L*γ* power (change of −0.26 dB) from baseline to motor onset in an example electrode with significant $MI_{z}$ during the baseline interval that decreases substantially with motor onset. See Extended Data Figure 5-1 for an example electrode's power spectra with the estimated aperiodic component during each interval.

L*γ* amplitude has been shown to decrease with movement in M1 (Igarashi et al., 2013). Since modulations in the band activity defining phase can modulate PAC (Aru et al., 2015; Nadalin et al., 2019), a simpler explanation for our findings is that the decrease in L*γ*–H*γ* PAC reflects decreased L*γ* amplitude. Although we cannot completely exclude this possibility, multiple pieces of evidence make it unlikely. Primarily, we utilized a modified GLM method that accounts for the amplitude of the band defining phase when estimating PAC strength, thus minimizing the effect of L*γ* amplitude on the estimated L*γ*–H*γ* PAC (Nadalin et al., 2019). While the modulation index method does not directly account for L*γ* amplitude, we observed an increase in L*γ*–H*γ* $MI_{z}$ with movement despite a corresponding decrease in L*γ* activity in some electrodes (Fig. 5*a*; Jensen et al., 2016). Additionally, in some electrodes, we observed little to no change in L*γ*–H*γ* $MI_{z}$ with movement despite a corresponding decrease in L*γ* activity (Fig. 5*b*). In some electrodes with relatively strong L*γ* activity, we observed no significant L*γ*–H*γ* $MI_{z}$ (Fig. 5*c*). In other electrodes, we observed decreases in L*γ*–H*γ* $MI_{z}$ with movement despite little change in L*γ* activity (Fig. 5*d*). Moreover, we observed elevated L*γ* activity relative to the estimated aperiodic component in most electrodes with significant PAC (Extended Data Fig. 4-1).

10.1523/ENEURO.0163-23.2023.f5-1 Figure 5-1 Power spectra from an example electrode during the word-reading task. a Increase in Lγ (40-50 Hz, horizontal dashed black lines) power relative the estimated aperiodic component during the baseline interval. b Increase in Lγ (40-50 Hz, horizontal dashed black lines) power relative the estimated aperiodic component during the movement interval. Download Figure 5-1, TIF file.

Beyond providing insight on network dynamics, PAC may also have practical clinical applications. In patients with Parkinson's disease, a condition that includes bradykinesia, rigidity, and freezing of gait, there is exaggerated *β*–*γ* PAC in motor regions that decreases with therapeutic deep brain stimulation (DBS) of the subthalamic nucleus, suggesting that elevated *β*–*γ* PAC reflects a motor-suppressed state (De Hemptinne et al., 2013, 2015; Yin et al., 2022). Further, exaggerated *α*–*γ* PAC has been found in patients with essential tremor (Kondylis et al., 2016). In both disorders, this abnormal PAC was more widespread spatially than in people without movement disorders. In addition to being a biomarker for symptom severity in Parkinson's disease, *β*–*γ* PAC has potential use as a feedback signal for closed-loop (adaptive) DBS (De Hemptinne et al., 2013, 2015; Swann et al., 2015; Qasim et al., 2016; Habets et al., 2018; Malekmohammadi et al., 2018; Bouthour et al., 2019; Hwang et al., 2020). In addition to observing L*γ*–H*γ* sometimes being the predominant type of PAC, we noted that the pattern of modulation differed between *β*–H*γ* and L*γ*–H*γ* PAC with motor onset (Extended Data Fig. 5-1). Moreover, our results suggest that L*γ*–H*γ* PAC contains different information about motor behavior activation than the well-described modulations in *β* and H*γ* band powers (Fig. 5). Since our results suggest that modulation of L*γ*–H*γ* PAC is a unique motor-related phenomenon, it would be interesting to investigate its relative strength and modulation pattern in patients with movement disorders. Once characterized in this population, L*γ*–H*γ* PAC, along with other markers related to motor activity and activation, could potentially be used to develop a more sophisticated adaptive DBS scheme via a multi-input control system.

Additionally, *β*–H*γ* PAC has been used to detect seizures during invasive monitoring for epilepsy surgery (Edakawa et al., 2016), and δ–L*γ* PAC was able to identify the postictal generalized EEG state that tends to present in patients at risk for sudden unexpected death in epilepsy (Grigorovsky et al., 2020). Thus, investigating L*γ*–H*γ* PAC in this patient population might possibly provide another marker to improve seizure monitoring and predicting outcomes in epilepsy patients. Furthermore, abnormal PAC may represent promising neurophysiological markers of schizophrenia (Hirano et al., 2018; Won et al., 2018), obsessive compulsive disorder (Bahramisharif et al., 2016), and Alzheimer's disease (Etter et al., 2019). If these reports of abnormal PAC can be demonstrated to reliably correlate with symptom severity, they may be used to develop stimulation paradigms aimed at alleviating these symptoms in these often-debilitating psychiatric conditions. Additionally, several types of PAC, including L*γ*–H*γ* PAC, contain some information about speech that may be used for simple decoding tasks (Proix et al., 2022). Thus, there are a number of potential therapeutic applications for which L*γ*–H*γ* PAC may contribute to improved functional outcomes.

## Synthesis

Reviewing Editor: Mark Laubach, American University Decisions are customarily a result of the Reviewing Editor and the peer reviewers coming together and discussing their recommendations until a consensus is reached. When revisions are invited, a fact-based synthesis statement explaining their decision and outlining what is needed to prepare a revision will be listed below. The following reviewer(s) agreed to reveal their identity: Tanuj Gulati. Note: If this manuscript was transferred from JNeurosci and a decision was made to accept the manuscript without peer review, a brief statement to this effect will instead be what is listed below.

Please see the reviewer's comments below and revise your manuscript accordingly. Also, please consider adding a visual abstract, which helps with attracting readers to your paper. Thank you for sending your study to eNeuro.

Reviewer #1

This manuscript by Nie et al. studies phase-amplitude coupling (PAC) between the phase of low gamma (40-50 Hz) and amplitude of high gamma (70-200 Hz) frequency bands in local-field potentials (LFPs) and electrocorticography (ECoG) recordings performed in the sensorimotor cortex in monkeys and sensorimotor and premotor cortices in humans, respectively, during different motor behaviors with varying complexity. In monkeys they have used a reaching task, and in humans, a finger flexion, or a speaking task. The authors have studied the changes in PAC during baseline and around task-onset using modulation index (MI) and a generalized linear model (GLM) framework. They have found that PAC is higher in baseline period and significantly reduces around task-onset. They have also found that this coupling was present in premotor and sensorimotor cortices of both species. Overall, the study addresses an important question about network dynamics during motor behaviors. The finding is striking across species and supported by appropriate analysis. The manuscript is well-written and well-organized. It is also timely with the recent reports of hand-knob area showing speech modulation. I list below some concern that can help improve the manuscript.

• One of the concerns is that can they also look at high gamma amplitude's coupling with the phase of beta. Since beta desynchronization is such a well-documented phenomena, and it is close to the low-gamma range, it can also be tested as marker of network dynamics.

Minor concerns

• What was the purpose of random hold time (Ln 158; 0.2-0.4 s) in monkey center out task? If there is no overshooting, monkey can just hold it and wait for the reward.

• MI can be italicized. It appears very similar to M1.

• Draw and outline of central sulcus in Fig. 3g, I and Fig 4 a,g and i for easy visualization.

• Ln 474: Make Fig 4i 's 'i' small case.

• Ln 513: Calling high gamma non-oscillatory may not be required. Please remove it or add a sentence justifying this.

Reviewer #2

The research manuscript investigates the presence and modulation of phase-amplitude coupling (PAC) between low-frequency (Lγ) and high-frequency (Hγ) brain oscillations during motor behaviors in monkeys and humans. The study demonstrates that Lγ-Hγ PAC is widespread across different motor regions and species, with consistent modulation patterns during motor behaviors. Overall this is an intersting effort as it combines data from non-human primates and invasive intracranial recordings from human subjects. The results suggest that modulation of Lγ-Hγ PAC is a motor-related phenomenon indicative of underlying network dynamics involved in the activation of motor behaviors.

A few critiques:

- Introduction is good in many respects but lacks discussion on the potential relevance of phase-amplitude coupling to motor behaviors.

- One large general question is that the gamma PAC seems to be more or less everywhere during active behaviors versus rest. So unclear to me whether this truly has functional relevance versus merely being a mathematical property of the recorded signals. The authors seem to acknowledge this potential limitation and comment thoughtfully on it. High frequency broadband activation and beta suppression occur during task execution if recorded from the appropriate contact. How do we know that the gamma PAC observation carries greater information content or more specific information content versus these other better-known components of the signals.

- The methods section should provide more details about the experimental setup, data collection, and analysis techniques used.

- The results section includes numerous abbreviations, figures, and statistical findings; the interpretation and significance of these results could be explained more explicitly. Excessive use of abbreviations compromises readability - please try to summarize and simplify. Results in some spots might benefit from a summary table rather than typing out all the statistics in narrative form.

- The discussion would benefit from more content on the potential implications of the findings and their significance in the broader context of motor control research.

- The writing would benefit from better overall structural organization to enhance readability. The combination of animal and human studies, including hand and voice recordings complicates the overall presentation.
