# Supplementary material for: High-Gamma Activity Is Coupled to Low-Gamma Oscillations in Precentral Cortices and Modulates with Movement and Speech
Source: eNeuro. 2024 Feb 9;11(2):ENEURO.0163-23.2023. doi: 10.1523/ENEURO.0163-23.2023 (PMC10867721; doi:10.1523/ENEURO.0163-23.2023)
Supplement: Table 1-1 — Participant demographics. Download Table 1-1, DOCX file. [file eneuro-11-ENEURO.0163-23.2023-s002.docx]

**Table 1-1.** Participant demographics

| Participant | Sex | Age (years) |
| --- | --- | --- |
|  |  |  |
| FM1 | M | 48 |
| FM2 | M | 33 |
| FM3 | M | 60 |
| FM4 | M | 30 |
| FM5 | M | 34 |
| WR1 | M | 57 |
| WR2 | M | 72 |
| WR3 | M | 43 |
| WR4 | M | 61 |
| WR5 | F | 45 |
| WR6 | M | 54 |
| WR7 | M | 50 |
