# Supplementary figures and images for: High-Gamma Activity Is Coupled to Low-Gamma Oscillations in Precentral Cortices and Modulates with Movement and Speech
Source: eNeuro. 2024 Feb 9;11(2):ENEURO.0163-23.2023. doi: 10.1523/ENEURO.0163-23.2023 (PMC10867721; doi:10.1523/ENEURO.0163-23.2023)

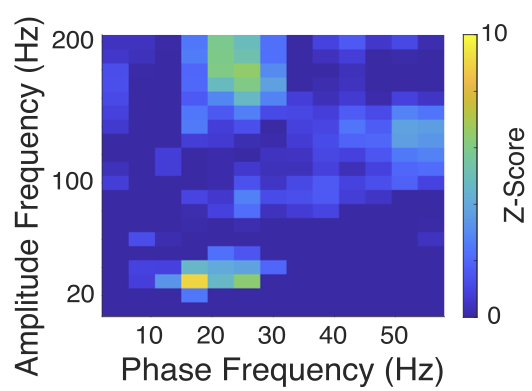

Supplement: Figure 2-1 — Comodulogram of example electrode demonstrating other types of phase-amplitude coupling (β-Hγ PAC here) in addition to Lγ-Hγ PAC during the reaching task. Download Figure 2-1, TIF file. [file eneuro-11-ENEURO.0163-23.2023-s003.tif]

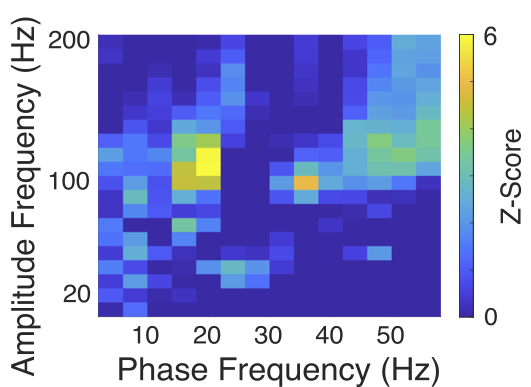

Supplement: Figure 3-1 — Comodulogram of example electrode demonstrating other types of phase-amplitude coupling (θ-Hγ, μ/α-Lγ, μ/α-Hγ, and β-Hγ PAC here) in addition to Lγ-Hγ PAC during the finger-flexion task. Download Figure 3-1, TIF file. [file eneuro-11-ENEURO.0163-23.2023-s004.tif]

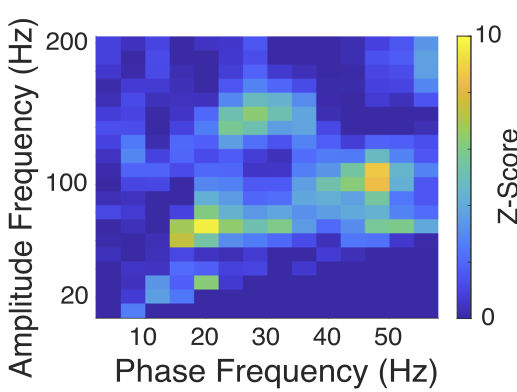

Supplement: Figure 4-1 — Comodulogram of example electrode demonstrating other types of phase-amplitude coupling (μ/α-Hγ, β-Lγ, and β-Hγ PAC here) in addition to Lγ-Hγ PAC during the word-reading task. Download Figure 4-1, TIF file. [file eneuro-11-ENEURO.0163-23.2023-s005.tif]

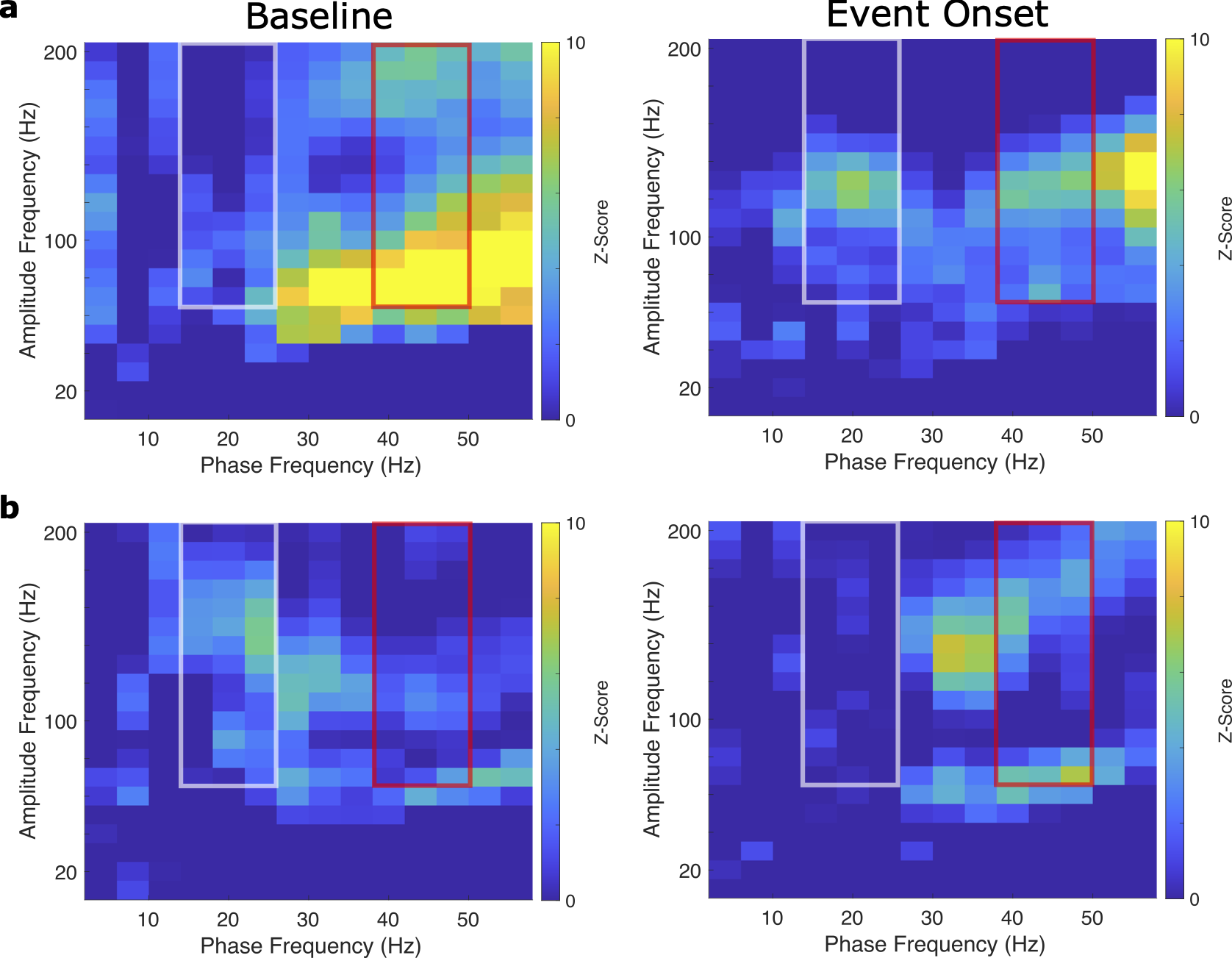

Supplement: Figure 4-2 — Example comodulograms from the word-reading task during the baseline and event-onset (voice-onset) intervals. These demonstrate different modulation patterns of β-Hγ and Lγ-Hγ PAC, as measured by the mean z-scored modulation index () across the wide PAC bands encompassing both β (16-24 Hz, white) and Lγ (40-48 Hz, red) with Hγ (70-200 Hz). a Increase β-Hγ PAC from baseline ( MIz¯ of 0.87) to event-onset ( MIz¯ of 1.48) intervals despite decrease in Lγ-Hγ PAC from baseline ( MIz¯ of 6.03) to event-onset ( MIz¯ of 1.94) intervals. b D MIz¯ecrease in β-Hγ PAC from baseline ( MIz¯ of 2.06) to event-onset ( MIz¯ of -0.13) intervals despite increase in Lγ-Hγ PAC from baseline ( MIz¯ of 0.68) to event-onset ( MIz¯ of 1.94) intervals. Download Figure 4-2, TIF file. [file eneuro-11-ENEURO.0163-23.2023-s006.tif]

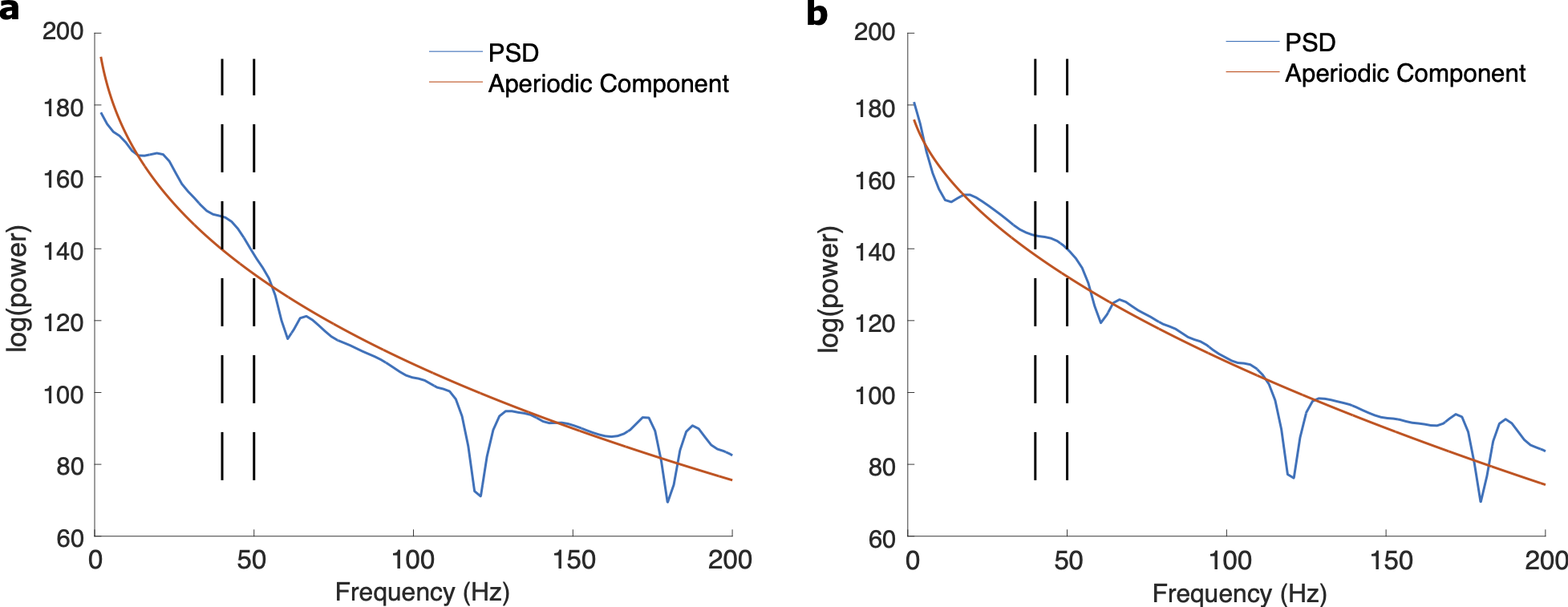

Supplement: Figure 5-1 — Power spectra from an example electrode during the word-reading task. a Increase in Lγ (40-50 Hz, horizontal dashed black lines) power relative the estimated aperiodic component during the baseline interval. b Increase in Lγ (40-50 Hz, horizontal dashed black lines) power relative the estimated aperiodic component during the movement interval. Download Figure 5-1, TIF file. [file eneuro-11-ENEURO.0163-23.2023-s007.tif]
